# Autologous Whole-Blood Versus Corticosteroid Local Injection in Treatment of Plantar Fasciitis: A Randomized Single Blind Placebo-Controlled Study

**DOI:** 10.7759/cureus.45588

**Published:** 2023-09-20

**Authors:** Nikhil Digra, Anurag Beri, Shashank Sharma, Ramkrishna Verma

**Affiliations:** 1 Orthopedics, Government Medical College and Hospital, Amritsar, IND

**Keywords:** autologous whole-blood, visual analog scale, placebo, methylprednisolone, corticosteroid, plantar fascitis

## Abstract

Background: Autologous whole-blood intralesional injection has attracted interest as a possible means of treatment for chronic plantar fasciitis. We contrasted its effectiveness with that of corticosteroids, which have a longer history of success in treating tendinopathies such as plantar fasciitis. In order to monitor the disease's progress naturally, we also compared them with a placebo.

Methods: Sixty clinically diagnosed patients were taken up for intralesional injection of autologous whole blood (AWB), corticosteroid, and normal saline as placebo mixed with 2 mL of lignocaine after dividing them into three groups. Two doses were given and followed up in the third, sixth, and 12th weeks. The evaluation was done according to the visual analog scale (VAS) and the number of tablets of paracetamol (PCM) 500 mg consumed for the following period.

Results: When compared to the placebo group in the third, sixth, and 12th weeks, the corticosteroid group exhibited a significant improvement with a p<0.001 in the VAS score, whereas the autologous whole blood group showed no meaningful difference. When compared to the placebo group at the sixth and 12th weeks, the AWB group's VAS score showed a statistically significant difference with a p>0.001. At each follow-up, the placebo group consumed more analgesics than the corticosteroid group, with a p<0.001. Only in the third week of follow-up did AWB demonstrate a statistically significant difference in PCM consumption as compared to the corticosteroid group.

Conclusion: Statistically significant improvement was seen in both the AWB group and corticosteroid group as compared to the placebo group. The corticosteroid group achieved earlier and superior relief of pain while AWB had a longer lasting effect. Therefore, study results indicate almost similar results in short-term studies.

## Introduction

One of the most widespread musculoskeletal conditions affecting the general population is plantar fasciitis. The histological results of degenerative rather than inflammatory alterations within the plantar fascia are consistent with the relationship of plantar fasciitis (PF) with advancing age. These degenerative results provide credence to the idea that PF results from repeated microtrauma brought on by protracted weight-bearing activities [1]. Additionally, the discovery that only a small number of inflammatory cells were present in samples taken from instances with chronic PF provided support for it [2]. This is similar to other chronic tendinopathies where the lesion has a predominance of fibroblasts, increased ground substance, vascularity, and loss of collagen continuity [3].

Diagnostic imaging is not frequently required because the diagnosis of PF is primarily based on clinical symptoms, such as tightness and pain in the heels. Imaging can be helpful in patients with recalcitrant PF to confirm the diagnosis and rule out other musculoskeletal conditions. Calcaneal osteophyte growth (heel spurs) on x-ray and thicker plantar fascia >4.5 mm on USG or MRI are characteristic imaging findings of PF [4-6].

The initial approach to treating chronic plantar fasciitis often involves a combination of conservative measures, including rest, application of ice packs, non-steroidal anti-inflammatory drugs (NSAIDs), and adjustments to footwear, such as incorporating arch supports [7-9].

The resolution of the condition often requires multiple sessions of modalities, such as ultrasonic waves, electrical stimulation, and phonophoresis, with approximately 90% of patients recovering through non-surgical care. The most effective and safe non-surgical treatment for plantar fasciitis remains an area of ongoing investigation, as local intra-lesional injections or surgical plantar fascial release may be considered when standard conservative approaches prove ineffective. Exploring options like corticosteroids, botulinum toxin, autologous whole-blood (AWB), and platelet-rich plasma (PRP) injections locally and within the lesion can be considered based on research that evaluates the pros and cons of these treatment modalities [10-12].

Following the success of intra-lesional AWB injections for tennis elbow in all of Edwards et al.'s patients, Dr. Barrett was the one who successfully attempted AWB injections for PF in the beginning [13,14]. It has been done for tendinopathies, such as patellar tendinopathy and lateral epicondylitis. This treatment was used for chronic PF since the pathogenesis in chronic tendinopathies is identical. AWB was chosen as the medium for injection due to its minimally traumatic application, low risk of immune-mediated rejection, ease of preparation and acquisition, affordability, and high level of patient acceptance. Both AWB and PRP contain platelets that are rich in growth factors that promote angiogenesis and enhance growth factor expression and cell proliferation. These substances aid in the healing of chronic wounds and can partially modify the damaged tissue [13-18].

Based on the findings of this investigation, we decided to use corticosteroids. First, a typical treatment for heel pain is the use of corticosteroids. Second, corticosteroid injection has been demonstrated to be helpful if substantial PF symptoms continue for longer than eight weeks under conservative treatment. In more than 90% of patients, recovery is anticipated within the first few days following the injection [10,19-21]. Since corticosteroids have a potent anti-inflammatory effect by inhibiting fibroblast proliferation, reducing the degeneration of collagen and mucopolysaccharides, and increasing the expression of ground substance proteins, it is advised to use them to reduce tissue edema over time, which has been shown in histologic studies of chronic PF patients [2,22-23].

The path to resolution for this condition typically entails undergoing multiple sessions of modalities such as ultrasonic waves, electrical stimulation, and phonophoresis. Usually, approximately 90% of patients who opt for non-surgical care experience a favorable recovery. However, the pursuit of the non-surgical treatment with the highest efficacy and safety remains an ongoing quest, as the optimal approach has yet to be definitively identified. In cases where the condition proves resistant to the aforementioned conservative therapy strategies, additional options come into consideration, including the possibility of local intra-lesional injections or even surgical plantar fascial release. These interventions may be explored when all other avenues have been exhausted. The evaluation of treatment modalities, such as corticosteroids, botulinum toxin, autologous whole-blood, and platelet-rich plasma injections at local and intra-lesional levels presents an avenue of potential investigation. A thorough analysis of existing research underscores both the advantageous aspects and the limitations of these various treatment choices, guiding healthcare professionals in making informed decisions tailored to the individual needs of patients.

A possible study constraint was avoided by contrasting the groups with a control group that received injections of normal saline diluted with 2% lignocaine as a placebo. This allowed us to more accurately assess the effectiveness of both intra-lesional AWB injection and corticosteroid as potential treatments for PF. The addition of a control group further demonstrated the disease's unaffected natural course. In clinical settings, patients frequently report excruciating pain upon stepping out of bed in the morning, after prolonged hours of deskbound activity.

## Materials and methods

This study was a prospective randomized controlled trial. It was done at Government Medical College and Guru Nanak Dev Hospital, Amritsar, India, a tertiary care center catering rural population from 2020 to 2021 after getting proper clearance from the Institutional Ethics Committee. Sixty patients of either sex were enrolled after briefing them on the procedure and getting valid written consent.

Inclusion criteria were patients from the age group of 18-65 years of either sex having heel pain for the last two weeks and clinically diagnosed cases of plantar fasciitis. Exclusion criteria were having a history of trauma or a fracture treated in the past, uncontrolled diabetes mellitus, and infection at the local site. The patients were divided into three groups by simple randomization - A, B, and C, each with 20 individuals. Group A patients were injected with 2 mL of autologous whole-blood drawn from the antecubital vein and mixed with 2 mL of 2% lignocaine. Group B patients were injected with 40 mg methylprednisolone acetate mixed with 2 mL of 2% lignocaine. Group C patients were injected with 2 mL of normal saline mixed with 2 mL of 2% lignocaine.

All the procedures were performed under sterile aseptic precautions after proper scrubbing and painting with a povidone-iodine solution. The location of greatest discomfort, which was at the level of the medial process of the calcaneal tuberosity, was palpated to determine the injection site using the walkover technique. The procedure was repeated after two weeks.

Post-procedure follow-up done at the third, sixth, and 12th weeks was accessed by visual analog scale where zero meant no pain and 10 meant maximum pain. Responses were grouped under very good (<25), good (26-50), fair (51-75), and poor (>76). Tablet paracetamol 500 mg was allowed on an SOS basis if patients experienced any breakthrough pain during the period and were noted using the diary method and was later statistically evaluated.

## Results

The sixty patients enrolled in the study had the following demographics shown in Table 1. All the groups were evaluated on the third, sixth, and 12th week according to the visual analog scale, as shown in Table 2.

**Table 1:** Demographics of the patients.

Demographics	Group	Mean age
Age (mean) 21 to 62 years	Group A	41.45 year
	Group B	45 year
	Group C	42.20 year
Sex	Males	55%
	Females	45%

**Table 2:** Evaluation of groups A, B, and C during the third, sixth, and 12th weeks with respect to VAS score. VAS: visual analog scale; AWB: autologous whole blood

VAS score		Poor		Fair		Good		Very good	
		No.	Age (%)	No.	Age (%)	No.	Age (%)	No.	Age (%)
AWB	3rd week	12	60.00	5	25.00	3	15.00	0	0.00
	6th week	0	0.00	8	40.00	10	50.00	2	10.00
	12th week	0	0.00	3	15.00	8	40.00	9	45.00
Corticosteroid	3rd week	6	30.00	9	45.00	5	25.00	0	0.00
	6th week	0	0.00	5	25.00	6	30.00	9	45.00
	12th week	0	0.00	0	0.00	13	65.00	7	35.00
Placebo	3rd week	18	90.00	2	10.00	0	0.00	0	0.00
	6th week	16	80.00	4	20.00	0	0.00	0	0.00
	12th week	11	55.00	8	40.00	1	5.00	0	0.00

The fact that this study's intra-lesional AWB injection considerably reduced pain levels as measured by VAS further supported the idea that AWB might promote healing in PF. Kirmani et al. in their study on 55 patients found similar findings with AWB and assessed with VAS at third and sixth-month follow-up. They came to the conclusion that AWB is a cost-effective therapy option for patients with plantar fasciitis who did not respond to conservative treatments because there was a significant decline in VAS score [24].

In a study by Vahdatpour et al., 34 patients with PF were tested using AWB and PRP, and it was discovered that pain scores decreased during the course of the trial in both the PRP and AWB groups with no significant differences. Additionally, fascia thickness as measured by USG decreased in both the PRP and AWB groups, demonstrating comparable efficacy of PRP and WB for the short-term therapy of chronic PF [25].

This study demonstrated that the corticosteroid group showed an early, sharp drop in the VAS and a plateau in average pain levels which was maintained for at least 12 weeks. Similar results were found using the VAS scale in a study conducted in 2015 by Saba and El-Sherif, who came to the conclusion that corticosteroids were a successful treatment for plantar fasciitis. They noted significant symptomatic relief at four weeks of follow-up and the disappearance of hypoechogenicity in both the ultrasound- and palpation-guided groups [26].

All the groups were statistically evaluated on the third, sixth, and 12th weeks as per their VAS score (Table 3). All the groups were statistically compared among themselves for p-values (Table 4).

**Table 3:** Statistical evaluation of the three groups among themselves, the third, sixth, and 12th weeks, according to VAS. AWB: autologous whole-blood; VAS: visual analog scale

Variables	AWB		Corticosteroid		Placebo	
	Mean	SD	Mean	SD	Mean	SD
3rd week	74.350	20.568	66.100	19.037	85.550	6.778
6th week	47.100	16.704	32.500	23.853	84.200	10.018
12th week	28.050	20.618	31.000	13.002	74.700	12.994

**Table 4:** Comparison of the three groups among themselves, the third, sixth, and 12th weeks, according to their p-values. AWB: autologous whole-blood

Follow-up	Groups compared	p-Value
3rd week	AWB versus corticosteroids	0.268
	Corticosteroid versus placebo	0.001
	AWB versus placebo	0.093
6th week	AWB versus corticosteroids	0.032
	Corticosteroid versus placebo	0.001
	AWB versus placebo	0.001
12th week	AWB versus corticosteroids	0.829
	Corticosteroid versus placebo	0.001
	AWB versus placebo	0.001

These results lead us to conclude that intra-lesional AWB injection and corticosteroid are superior to placebo in terms of efficacy and performance and may be utilized as an injectable alternative for PF. Additionally, it shows that patients who had corticosteroid injections likely improved more significantly and more quickly than those who did not.

The results of group comparisons between the third and sixth weeks, the sixth and 12th weeks, and the third and 12th weeks are summarized in Table 4. The results demonstrated that patients who had a corticosteroid injection considerably improved more than those who received an AWB injection or were in the control group. However, in the third week of follow-up, the corticosteroid and control groups showed the most noticeable difference. However, to a lesser extent, improvement was also seen in the control group, a finding that would support the theory that the condition is self-limiting. Similar results were reported by three placebo-controlled randomized controlled studies, which showed that corticosteroid injections reduced PF pain more effectively than a placebo [2,19].

Although intralesional AWB injection is not as effective as corticosteroid, it may be utilized as an injectable alternative for PF in order to get long-term pain relief and drastically change the disease's natural course. Consumption of tablet paracetamol 500 mg as an analgesic was noted as shown in Table 5. Table 6 shows the statistical consumption of tablet paracetamol 500 mg, between the groups.

**Table 5:** Consumption of analgesic, tabulated in the third, sixth, and 12th weeks. AWB: autologous whole-blood

Follow-up	AWB		Corticosteroid		Placebo	
	Mean	SD	Mean	SD	Mean	SD
3rd week	15.100	3.596	9.300	4.473	17.150	2.033
6th week	7.200	3.053	4.000	2.317	15.500	2.5236
12th week	8.750	3.537	4.400	1.957	22.550	8.306

**Table 6:** Compassion between the groups according to analgesics consumed. AWB: autologous whole-blood

Follow-up	Groups compared	p-Value
3rd week	AWB versus corticosteroids	0.001
	Corticosteroid versus placebo	0.001
	AWB versus placebo	0.165
6th week	AWB versus corticosteroids	0.001
	Corticosteroid versus placebo	0.001
	AWB versus placebo	0.001
12th week	AWB versus corticosteroids	0.033
	Corticosteroid versus placebo	0.001
	AWB versus placebo	0.001

These results showed that, in comparison to the AWB group and the placebo group, the benefits of corticosteroids are more effective and manifest earlier in terms of pain alleviation and analgesic usage. The placebo group consumed analgesics at a considerably higher rate. At the conclusion of the study, it was discovered that AWB and corticosteroid both consumed an equal number of analgesics and performed statistically significantly better than the placebo group, making them both viable therapy options.

## Discussion

According to Li et al.'s 2015 meta-analysis, corticosteroid injections may temporarily reduce pain, but their effectiveness may diminish with time. To further assess the effectiveness, a study with long-term follow-up was also required [27].

When comparing the effects of AWB and corticosteroid injections on epicondylopathy or plantar fasciopathy, Tsikopoulos et al. conducted a systemic review and meta-analysis of nine randomized controlled trials (RCTs) in 2015. They came to the conclusion that corticosteroids were only slightly superior to AWB in relieving pain in plantar fasciopathy at two to six weeks [28].

Similar outcomes were observed in a trial by McMillan et al., which demonstrated that dexamethasone had a good treatment response when compared to the placebo [2]. The results of the current study indicated almost the same pattern; the corticosteroid group initially showed a dramatic decline in VAS and then quickly achieved a plateau in average pain levels, which was sustained for at least 12 weeks. According to a 2019 study by Liaqat et al. of 180 patients, the group treated with corticosteroids had significantly higher mean pain scores at 12 weeks post-treatment than the group treated with AWB. AWB was found to be superior to corticosteroid injection in PF patients after three months of follow-up [29]. Similar outcomes were observed by Majeed et al. in 2019, who evaluated 100 patients' VAS scores and discovered that corticosteroid injection considerably reduced PF pain more than AWB injection [30].

We discovered that AWB injection considerably decreased pain and discomfort in patients with plantar fasciitis without causing any negative side effects. When compared to corticosteroid injection (methylprednisolone 40 mg), which provided superior and early short-term relief of pain from plantar fasciitis with no potential risk of complications, AWB injection was simple to obtain, prepare, and carry out, and it was easy to perform. It is straightforward, reasonably affordable, efficient, and easily accessible, and it has a longer track record with fewer instances of problems.

We also found that the placebo injection of normal saline was not effective in significantly decreasing the VAS score in earlier follow-ups, but still, the group showed some relief in VAS score although it was along with higher consumption of analgesics when compared to other groups. The finding may signify the self-resolving nature of the disease and some role of tablet paracetamol in alleviating the suffering.

## Conclusions

At the end of the trial, there was no difference between the groups that got corticosteroids and autologous whole-blood, proving that both treatments were equally effective for treating plantar fasciitis. Therefore, the injection of corticosteroids should be the first line of treatment for plantar fasciitis as demonstrated in previous studies, with the injection of autologous whole-blood being a viable alternative for effective treatment of plantar fasciitis.

The study was limited in the number of patients enrolled in each group and shorter follow-up duration with fixed intervals. Hence a further multicentric observer-blind study is required with a larger sample size in each group and with a longer follow-up duration for substantiating the findings in this study.
